# Cardiorespiratory Fitness in the Prevention and Management of Cardiovascular Disease

**DOI:** 10.31083/j.rcm2311382

**Published:** 2022-11-15

**Authors:** Michael J. LaMonte

**Affiliations:** ^1^Department of Epidemiology and Environmental Health, School of Public Health and Health Professions, University at Buffalo – SUNY, Buffalo, NY 14214, USA

**Keywords:** heart disease, exercise, physical activity, maximal oxygen uptake, risk assessment, exercise prescription, prognosis

## Abstract

Cardiovascular disease (CVD) is the leading cause of death among adults in the U.S. and elsewhere. Variation in the presence, severity, and control of major modifiable risk factors accounts for much of the variation in CVD rates worldwide. Cardiorespiratory fitness (CRF) reflects the integration of ventilation, circulation, and metabolism for the delivery and utilization of oxygen in support of dynamic aerobic physical activity. The gold standard measure of CRF is maximal oxygen uptake. Because the primary factor underlying differences in this measure between individuals is maximal cardiac output, it can serve as a clinical indicator of cardiac function. Higher CRF is associated with favorable levels of major CVD risk factors, lower prevalence and severity of subclinical atherosclerosis, and lower risks of developing both primary and secondary clinical CVD events. The beneficial associations between CRF and CVD are seen in women and men, older and younger adults, in those with multiple coexisting risk factors or prior diagnosis of CVD. Exercise training and regular physical activity of at least moderate intensities and volumes improves CRF in adults, and improvements in CRF are associated with lower risks of subsequent CVD and mortality. Routine assessment of CRF in primary care settings could enhance individual-level CVD risk assessment and thereby guide implementation of appropriate measures to prevent future clinical events.

## 1. Introduction

Cardiorespiratory fitness (CRF) is a strong, independent predictor of future cardiovascular clinical events and mortality [1, 2, 3]. When measured carefully in a clinical setting, CRF has been more strongly associated with cardiovascular outcomes than other exercise test responses including patient symptoms, electrocardiographic and hemodynamic factors [4, 5]. CRF reflects assimilation of anatomical, physiological, biochemical, and neuromuscular inputs that represent far more than an individual’s exercise habits. As such, CRF is considered a hallmark of aging resiliency [6]. While CRF is a recognized biomarker of physical function and cardiovascular health, it is not currently included with other established risk factors, such as blood pressure or cholesterol, in clinical practice guidelines for cardiovascular disease (CVD) risk assessment. Routine assessment of CRF in primary care settings not only will provide the physician with valuable clinical data on their patient’s health status, but could potentially foster health behavior changes to improve CRF knowing that it is part of the annual health record along with body weight, blood pressure, and other vital signs [5].

### Background

Beginning around 1950, numerous scientific publications have documented the relationship of physical activity (PA) and cardiorespiratory fitness (CRF) with cardiovascular health and disease [1, 2, 3]. Not surprising, these investigations have differed substantially with respect to study population, size and design, the cardiovascular outcome investigated, and the type of assessment used to measure PA or CRF. Nevertheless, the overwhelming finding among the studies of higher quality (e.g., adequate sample size and statistical power, well-documented quantification of PA or CRF) has been consistency in cardiovascular health benefits associated with higher levels of activity and fitness. Because of their relatively high prevalence at the population level, the population attributable risk (e.g., percentage of disease cases attributed to a risk factor) for all-cause and cardiovascular mortality associated with sedentary behavior and low CRF is comparable to that of other major modifiable cardiovascular risk factors such as hypercholesterolemia, hypertension, and smoking [7, 8]. Table 1 (Ref. [7]) illustrates this showing population attributable risks of CVD mortality for low CRF and other modifiable CVD risk factors in adults ages 18–98 years who were without known CVD or cancer and followed an average of 17 years [7]. Assuming the association between CRF and CVD mortality is causal, if all individuals with low CRF improved to even moderate levels of CRF then 1 in 4 CVD deaths among women and men each in this population might have been avoided. Only hypertension accounted for a high proportion of deaths therein. While population attributable risk is a theoretical estimate, it does bring into context the force an exposure exerts on population health which depends on the amount of exposure and the strength of its association with CVD [9]. Because of the relatively high prevalence of low CRF and its strong association with CVD mortality, the potential population effect for delaying CVD mortality through increases in CRF is considerable. Indeed, leading authorities assert that low CRF could be the biggest public health threat of the 21st century [10] and, as such, CRF should be considered a standard clinical vital sign assessed regularly and targeted for modification just like other conventional risk factors monitored for cardiovascular health [11]. Because measured CRF is less prone to misclassification resulting from response biases or behavioral reactivity as compared to self-reported or directly monitored PA habits, CRF may better reflect the adverse consequences of a sedentary lifestyle [12]. This might not only be because due to more reliable measurement than reported PA levels, but also because CRF may better reflect the combined effects of genetics and behavior in determining an individual’s health status.

**Table 1.** **Population attributable risk (PAR%) of CVD mortality**.

	Men (N = 40,872)			Women (N = 12,943)		
Risk Factor	$P_{e}$	HR (95% CI)	PAR%	$P_{e}$	HR (95% CI)	PAR%
Low CRF	42.9	2.78 (2.29, 2.89)	29.9	41.2	3.32 (2.31, 4.78)	28.8
Self-reported sedentary	52.7	1.27 (1.11, 1.42)	11.2	51.9	1.36 (0.93, 1.99)	13.7
Obesity	19.3	2.08 (1.81, 2.39)	9.9	13.7	3.01 (1.82, 4.97)	9.2
Current smoker	25.5	1.51 (1.33, 1.72)	8.6	19.1	1.61 (1.03, 2.51)	7.2
Hypertension	56.9	2.23 (1.99, 2.49)	31.4	50.4	3.24 (2.29, 4.57)	34.8
Hypercholesterolemia	43.2	1.68 (1.51, 1.88)	17.4	38.2	1.68 (1.18, 2.39)	15.5
Diabetes	15.8	2.26 (1.94, 2.62)	8.8	9.2	3.55 (1.96, 6.44)	6.6

HR (95% CI) adjusted for age and examination year. $P_{e}$, prevalence of exposure in decedents; HR, hazard ratio; CI, confidence interval. PAR% calculated as $P_{e}$ (1 - 1/HR). *Adapted from LaMonte MJ. Epidemiology of Cardiovascular Disease. In: JL Durstine, GE Moore, MJ LaMonte, BA Franklin (eds.) Pollock’s Textbook of Cardiovascular Disease and Rehabilitation (pp. 9–22). Human Kinetics: Champaign, IL. 2008. [7].*

The objective of this report is to overview the cardiovascular health benefits associated with greater levels of CRF in both primary and secondary CVD prevention. Key points will be illustrated using results from selected individual studies that are frequently cited in consensus statements and systematic reviews. Streams of evidence from both observational and experimental studies will be discussed when possible.

## 2. Defining Cardiorespiratory Fitness

**Fig. 1.**
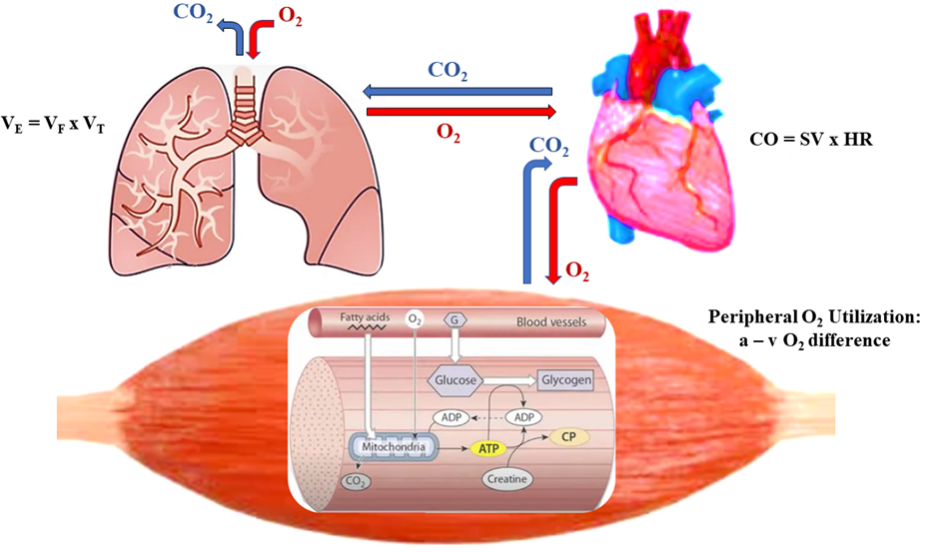
**Ventilation → Circulation → Metabolism**. $V_{E}$, minute ventilation; $V_{F}$, ventilatory frequency; $V_{T}$, tidal volume; ${\mathrm{CO}}_{2}$, carbon dioxide; $O_{2}$, oxygen; CO, cardiac output; SV, stroke volume; HR, heart rate; a-${\mathrm{vO}}_{2}$, arterial-venous oxygen difference.

CRF is one of several physiological attributes collectively referred to as physical fitness, the other attributes being body composition, muscular strength and endurance, agility, balance, and reaction time [13]. CRF (also referred to as cardiovascular, cardiopulmonary, aerobic, or endurance fitness) reflects the ability of the cardiopulmonary system to supply oxygen to working skeletal muscles, and of muscles to effectively utilize oxygen to support performance of dynamic PA [13]. CRF, thus, reflects an integrated system that links *ventilation* ($O_{2}$ intake, ${\mathrm{CO}}_{2}$ emission), *circulation* ($O_{2}$ delivery, ${\mathrm{CO}}_{2}$ removal), and *metabolism* ($O_{2}$ utilization, ${\mathrm{CO}}_{2}$ production) as depicted in Fig. 1. The gold standard measurement of CRF is the maximal oxygen uptake ($\dot{V}\,O_{2\,m\,a\,x}$) defined as the rate of oxygen utilization per minute standardized per kilogram body weight (e.g., mL $O_{2}$ •${\mathrm{kg}}^{-1}$•$\min^{-1}$) [4, 13, 14]. $\dot{V}\,O_{2\,m\,a\,x}$ is a product of stroke volume × heart rate × arterio-venous $O_{2}$ difference, where stroke volume is determined by left ventricular diastolic relaxation efficiency, myocardial and pericardial compliance; heart rate is determined by sympathetic nervous system outflow; and arterio-venous $O_{2}$ difference is determined by skeletal muscle energetic efficiency [15, 16]. Variation in $\dot{V}\,O_{2\,m\,a\,x}$ across populations generally results from differences in maximal cardiac output, which is determined by maximal stroke volume and heart rate. Factors influencing CRF include age, sex, health status, and genetics; however, the principal modifiable factor is habitual PA level. CRF responses to a standardized dose of aerobic exercise training vary widely among individuals, and the observed heterogeneity is not random but rather aggregates in families through both genetic and environmental components [17]. Nevertheless, in most individuals and particularly among those who are sedentary, increases in PA result in increases in CRF, whereas CRF declines soon after cessation of PA [13]. Thus, CRF has been used as an objective surrogate measure of recent PA patterns [18].

In clinical settings CRF is often used as a measure of exercise tolerance or physical functioning capacity expressed as metabolic equivalent (METs) or *multiples of resting oxygen uptake * [4, 19]. One MET (resting oxygen uptake) is assumed over a wide adult age range to be 3.5 mL $O_{2}$ •${\mathrm{kg}}^{-1}$•min$\min^{-1}$. An individual with a 5 MET level of CRF is capable of maximal exertion equivalent to 5-times that of resting energy expenditure (e.g., 17.5 mL $O_{2}$•${\mathrm{kg}}^{-1}$•$\min^{-1}$); a 10 MET level of CRF is 10-times resting energy expenditure (e.g., 35 mL $O_{2}$ •${\mathrm{kg}}^{-1}$•$\min^{-1}$). In a clinical context, low maximal MET levels of CRF, such as ≤3 METs (e.g., inability to complete Stage I of the Bruce treadmill protocol), are used to identify patients with severe cardiac failure who qualify for heart transplantation [20], whereas a 10 MET maximal CRF (e.g., completion of Stage III of the Bruce protocol) identifies exceptionally good prognosis in patients with stable coronary artery disease regardless of number of diseased vessels or presence of exercise-induced ischemic electrocardiographic sequalae [21]. By contrast, elite athletes can have exceptionally high maximal MET levels of CRF, such as 17 METs (elite soccer), 22.5 METs (elite cycling) and 24.1 METs (elite distance running) [22, 23, 24]. Table 2 (Ref. [25]) gives sex- and age-specific expected maximal levels of CRF for apparently healthy adults without known CVD who completed symptom-limited maximal exercise treadmill testing as part of an elective preventive medical examination at the Cooper Clinic (Dallas, TX, USA) [25]. CRF is clearly inversely related with age and is higher in men than women at a given age. While it had long been thought that CRF declines by about 1% per year over the adult age range [26], recent longitudinal studies have shown that CRF does not decline linearly with age, but rather there are accelerations in loss of CRF beginning around age 60 and again thereafter [27, 28], a trend attributed in large to loss of lean body mass with aging [27]. The limited available data describing secular trends indicate an apparent increase in CRF among U.S. adults during the 1970s to about 1990 followed by a slight decline during the early 2000s [29, 30]. The latter observation parallels the declines among Swedish [31] and Canadian [32] adults during the first two decades of the 2000s.

**Table 2.** **Maximal CRF levels for apparently healthy men and women**.

Age (years)	Percentile	Men		Women	
		mL $O_{2}$/kg/min	METs	mL $O_{2}$/kg/min	METs
20–39	≤20th (low)	<36.4	<10.4	<28.7	<8.2
	20th–40th	36.4–40.9	10.4–11.7	28.7–32.9	8.2–9.4
	40th–60th	40.9–45.6	11.7–13.1	32.9–36.4	9.4–10.4
	60th–80th	45.6–50.4	13.1–14.4	36.4–40.9	10.4–11.7
	≥80th (high)	>50.4	>14.4	>40.9	>11.7
40–49	≤20th (low)	<34.7	<9.9	<26.6	<7.6
	20th–40th	34.7–37.8	9.9–10.8	26.6–29.8	7.6–8.5
	40th–60th	37.8–42.7	10.8–12.2	29.8–32.9	8.5–9.4
	60th–80th	42.7–47.3	12.2–13.5	32.9–37.8	9.4–10.8
	≥80th (high)	>47.3	>13.5	>37.8	>10.8
50–59	≤20th (low)	<29.8	<8.5	<23.5	<6.7
	20th–40th	29.8–34.7	8.5–9.9	23.5–26.6	6.7–7.6
	40th–60th	34.7–37.8	9.9–10.8	26.6–29.8	7.6–8.5
	60th–80th	37.8–43.1	10.8–12.3	29.8–33.6	8.5–9.6
	≥80th (high)	>43.1	>12.3	>33.6	>9.6
≥60	≤20th (low)	<25.2	<7.2	<20.3	<5.8
	20th–40th	25.2–29.8	7.2–8.5	20.3–23.5	5.8–6.7
	40th–60th	29.8–33.3	8.5–9.5	23.5–26.6	6.7–7.6
	60th–80th	33.3–37.8	9.5–10.8	26.6–30.1	7.6–8.6
	≥80th (high)	>37.8	>10.8	>30.1	>8.6

METs, metabolic equivalents; 1 MET = 3.5 mL $O_{2}$ uptake •${\mathrm{kg}}^{-1}$•$\min^{-1}$. *Adapted from Sui X, LaMonte MJ, Blair SN. American Journal of Epidemiology. 2007; 65: 1413–1423. [25].*

## 3. Measuring Cardiorespiratory Fitness

CRF can be measured using both submaximal and maximal exercise tests and a variety of testing modalities in laboratory and field settings [19, 33, 34]. Direct quantification of $\dot{V}\,O_{2\,m\,a\,x}$ (mL $O_{2}$ •${\mathrm{kg}}^{-1}$•$\min^{-1}$) through analysis of expired gas concentrations at maximal physical exertion is the gold standard measure of CRF. Upright maximal exercise testing using a calibrated motor-driven clinical treadmill or electronically-braked cycle ergometer is the preferred testing modality, with $\dot{V}\,O_{2\,m\,a\,x}$ being 10–25% higher during treadmill ergometry because of the larger skeletal muscle mass engaged and the premature termination of cycle tests because of localized leg muscle fatigue during a less familiar form of activity for many adults [34]. When direct measurement of $\dot{V}\,O_{2\,m\,a\,x}$ is not feasible, submaximal and maximal exercise tests can be used to estimate $\dot{V}\,O_{2\,m\,a\,x}$ based on achieved physiological responses (e.g., heart rate) or workloads (e.g., cycle ergometer watts; treadmill speed and grade). Maximal testing is more burdensome as it requires participants to reach an endpoint of volitional exhaustion and in some circumstances may require specialized medical equipment and trained personnel to ensure participant safety. Nevertheless, the sensitivity of estimated CRF is highest when maximal exertion (or near maximal, e.g., >85% age-predicted heart rate maximum and/or >17 on a 20 point Borg Rating of Perceived Exertion scale) is achieved, particularly when comparing repeated assessments (e.g., change in CRF) among populations. Several submaximal tests of CRF have been used in clinical and research settings including step tests (e.g., Harvard Step Test; Canadian Fitness Test), cycle ergometry tests (e.g., Astrand-Rhyming single-stage; YMCA multi-stage), treadmill tests (e.g., Taylor-U.S. Railroad Study single-stage; Pollock-Wilmore two-stage), and walk tests (e.g., Cooper 12-minute or 1.5 mile test; Rockport 1 mile test). When ergometer (e.g., treadmill, cycle, step) testing is utilized a key assumption underlying predicted $\dot{V}\,O_{2\,m\,a\,x}$ is that steady-rate metabolism (e.g., heart rate, ventilation) was achieved during each stage of the test. Furthermore, because stroke volume plateaus at relatively low work rates, the higher the steady-rate submaximal heart rate (hence, cardiac output) achieved during the final test stage the greater the accuracy in $\dot{V}\,O_{2\,m\,a\,x}$ prediction.

**Fig. 2.**
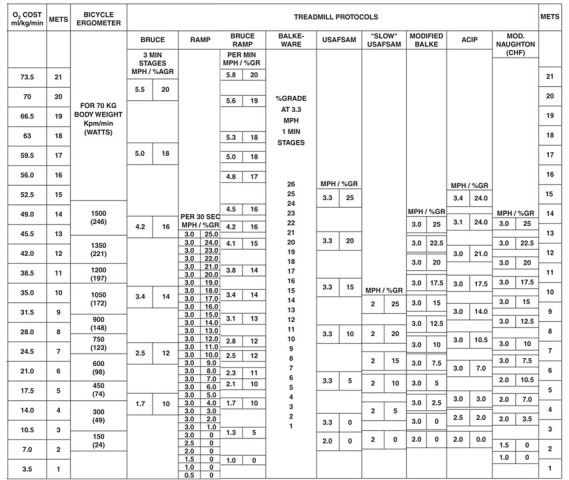
**Oxygen uptake according to various workloads and exercise testing modality**. $O_{2}$, oxygen; mL, milliliters; kg, kilogram; min, minute; METs, metabolic equivalents; kpm, kilopond meters; mph, miles per hour; sec, second; GR, grade; USAFSAM, United States Airforce School of Aerospace Medicine; ACIP, Asymptomatic Cardiac Ischemia Pilot. *Adapted from ACSM’s Guidelines for Exercise Testing and Prescription. 7th edn. Lippincott Williams & Wilkins: Philadelphia. 2006. [34].*

Shorter timed walk tests, such as the 400 meter and 6-minute walk, are readily used in clinical and epidemiological settings to assess physical function status as well as to predict $\dot{V}\,O_{2\,m\,a\,x}$ with reasonably high accuracy when compared against directly measured $\dot{V}\,O_{2\,m\,a\,x}$ (e.g., $R^{2}$ = 0.71–0.76; SEE <1.5 METs) [35, 36]. The choice of performance-based test to assess CRF will depend on available equipment and testing personnel, population being studied, participant burden and safety, and time and budget constraints. Non-exercise test prediction models also have been developed using a variety of demographic, lifestyle, and clinical factors for use when performance-based assessment is not feasible [37, 38]. Approaches previously used to assess CRF in studies on CVD incidence and prognosis include direct measurement of $\dot{V}\,O_{2\,m\,a\,x}$ [39, 40], maximal [41, 42] and submaximal [43, 44] treadmill and cycle ergometry, step testing [45], timed walk tests [46, 47], and non-exercise test equations [48, 49]. Fig. 2 (Ref. [34]) shows expected values of oxygen uptake associated with various testing modalities and workloads.

## 4. CRF and Development of CVD

**Fig. 3.**
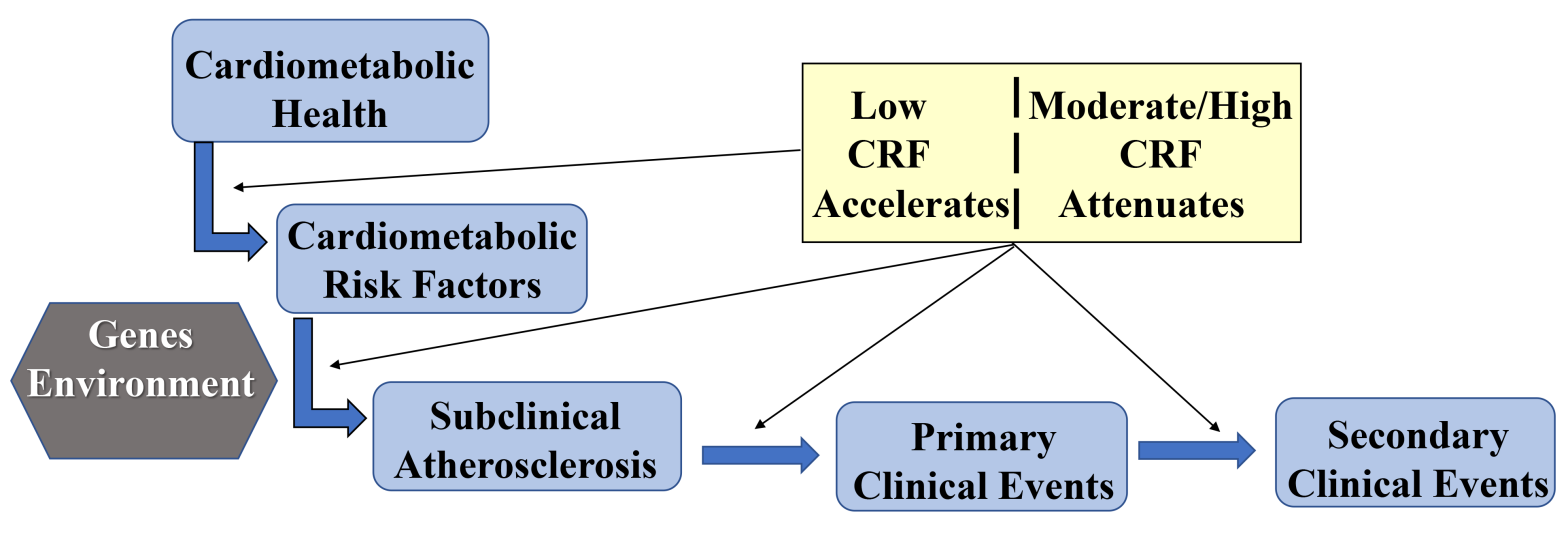
**Conceptual framework of CRF pathways to CVD prevention**.

Atherosclerosis, the underlying disease process of most CVD deaths in U.S. adults, is a complex process that starts early in life and progresses over decades in a subclinical state before manifestation of clinical CVD events in mid- to later life [7]. Interaction of environmental factors and individual-level susceptibility traits lead to development of major modifiable CVD risk factors which initiate formation of atherosclerotic lesions within the coronary arteries. If unchecked, the disease progresses and eventually presents clinically as angina, myocardial infarction, or sudden cardiac death. As illustrated in Fig. 3, there are several plausible pathways through which higher CRF impacts the initiation and progression of atherosclerotic CVD for both primary and secondary prevention of clinical CVD events. The following sections will briefly review evidence supporting this conceptual framework.

### 4.1 CRF and CVD Risk Factors

Variation in the presence, severity, and control of major modifiable CVD risk factors is a principal determinant of differences in CVD burden between populations [50, 51]. In the U.S. National Health and Nutrition Examination Survey between 2007 and 2018, trajectories for some modifiable risk factors (current smoking, leisure-time physical activity, serum total cholesterol) showed significant improvement whereas other risk factors (body mass index, dietary intake, blood pressure, serum glucose and hemoglobin A1c) worsened during the same time interval [52]. Variation in CVD risk factors was clearly evident according to subgroups defined by age (worse in older adults) and race and ethnicity (worse in black compared to white and Hispanic adults). While widespread use of pharmacotherapies to control major CVD risk factors is likely benefiting certain factors (e.g., lipids [53]), there remains a substantial burden of untoward risk factors in the population that will increase with an aging society and will translate into higher frequency of clinical CVD events if not brought into check [54]. Use of nonpharmacologic behavioral strategies to enhance risk factor control is, therefore, of high importance to preventive cardiology and public health.

#### 4.1.1 Risk Factor Prevalence

**Fig. 4.**
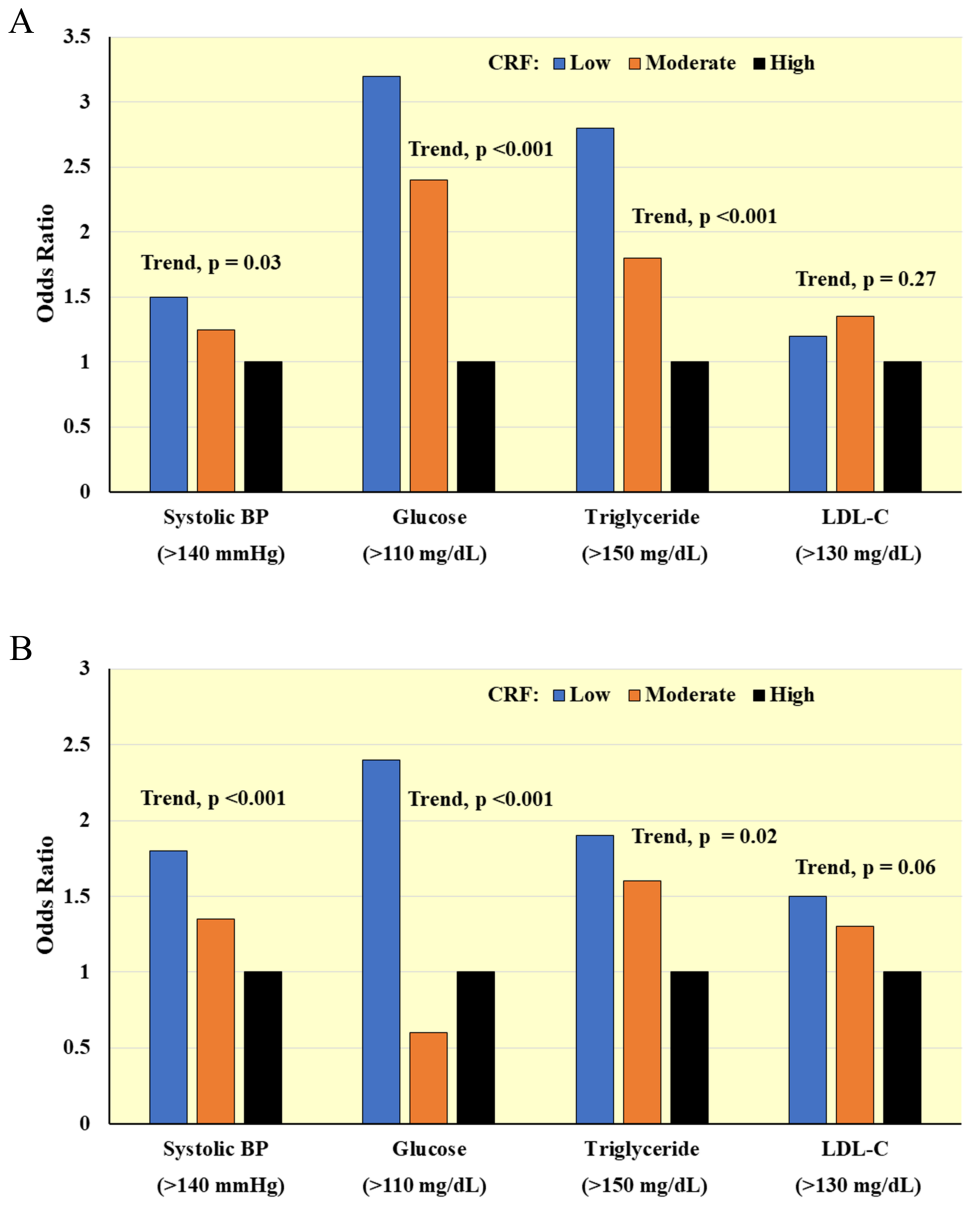
**Cross-sectional associations between CRF and clinically relevant CVD risk factors in (A) Men and (B) Women**. Odds ratios adjusted for age, percent body fat, smoking, and family history of CVD. BP, blood pressure; LDL-C, low-density lipoprotein cholesterol. *Adapted from LaMonte MJ et al., Circulation. 2000; 102(14): 1623–1628. [56].*

Higher CRF is associated with favorable levels of traditional CVD risk factors in cross-sectional studies of women and men with [55, 56] and without [56, 57, 58] existing CVD. Fig. 4 (Ref. [56]) shows inverse associations for CRF, assessed by maximal treadmill testing, with prevalence of clinically relevant CVD risk factors [56]. CRF is also associated with lower prevalence of coexisting cardiometabolic factors, *metabolic syndrome * [59, 60], in cohorts of middle-aged adults who were without known CVD at examination. The inverse association between CRF and prevalent metabolic syndrome is quite steep. Among 7104 women whose mean age was 44 years at the time of completing a symptom-limited maximal treadmill fitness test, the age, smoking, and exam year-adjusted prevalence of metabolic syndrome across incremental quintiles of CRF was 19%, 6.7%, 6.0%, 3.6%, and 2.3%, respectively (Trend, *p *< 0.01) [61]. A similar inverse pattern of association was seen among women within each decade category of age between 40 and 80 years.

CRF has also been associated with other biomarkers of cardiovascular health. Higher CRF is favorably related to pulse wave velocity [62] and coronary arterial diameter [63] and dilating capacity [64] (measures of arterial compliance), heart rate variability [65, 66] (measure of cardiac autonomic function), pericardial adipose deposition [67], and measures of cardiac size and function in adults residing in the community setting [68, 69, 70, 71, 72]. In one study that evaluated nitroglycerin-induced coronary vasodilation between runners and sedentary controls, there was a 2-fold greater increase in arterial cross-sectional area following nitroglycerin in runners that correlated (r = 0.68) strongly with $\dot{V}\,O_{2\,m\,a\,x}$ [64]. Cross-sectional studies in adults without known CVD indicated that higher CRF is associated with lower concentrations of inflammatory biomarkers high-sensitivity C-reactive protein and fibrinogen [73, 74], and cardiac troponin-T, a biomarker of subclinical myocardial injury [75]. In a study of 722 middle-aged men without known CVD, the multivariable-adjusted prevalence of elevated C-reactive protein (≥2.0 mg/L) was 50% and 18% in the lowest and highest CRF quintile, respectively (Trend, *p *< 0.001), a pattern of association observed even in men with abdominal obesity (waist girth ≥102 cm) [73]. In patients with existing atherosclerotic CVD or myocardial dysfunction, CRF tends to be inversely related with cardiac biomarkers [76, 77, 78]. Associations between CRF and CVD risk factors, cardiac biomarkers, and cardiac function have generally been independent of measures of adiposity [72, 73, 74] including directly measured visceral adiposity [79, 80]. Additional support for these cross-sectional observations comes from a recent meta-analysis showing moderate-to-vigorous intensity exercise training simultaneously improves CRF and several cardiometabolic biomarkers in apparently healthy adults and in those who are obese or have pre-existing health conditions [81].

#### 4.1.2 Risk Factor Incidence

**Fig. 5.**
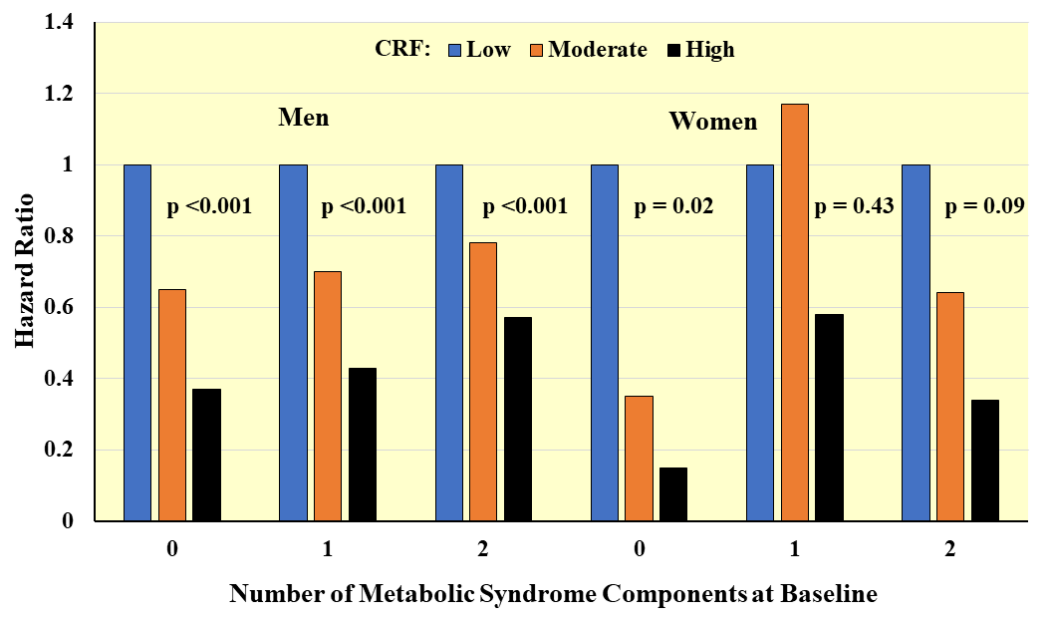
**Prospective associations between CRF and incident metabolic syndrome according to number of components at baseline**. Hazard ratios adjusted for age, exam year, BMI, smoking, alcohol, family history of CVD and diabetes. *Adapted from LaMonte MJ et al., Circulation. 2005; 112(4): 505–512. [83].*

Evidence that CRF levels are predictive of future development of clinically relevant risk factors would provide stronger inferences as compared to the cross-sectional findings reviewed above. However, few studies have examined prospective associations between a measure of CRF and incidence of cardiometabolic risk factors. One of the most comprehensive studies to date was reported in the CARDIA cohort where 2029 men and 2458 women, mean age 25 years at the time of maximal treadmill fitness testing, were followed for 15 years [82]. In analysis adjusted for demographic, anthropometric, family history, and self-reported PA information, the relative risk of incident hypertension, diabetes, metabolic syndrome, and elevated low-density lipoprotein cholesterol among non-obese participants was 1.21, 1.26, 1.28, and 1.08 (*p *< 0.05, all) for each 1-minute decrement (lower CRF) in treadmill test duration. Associations remained significant among obese individuals except for incident diabetes and hypercholesterolemia which attenuated to the null. Another 6-year prospective cohort study on 9007 men and 1491 women whose mean age was 44 years when completing maximal treadmill tests, observed inverse gradients (*p *< 0.001, each) in age-adjusted rates of incident metabolic syndrome over incremental tertiles of CRF in both women (10.4, 6.7, 3.1 per 1000) and men (44.1, 24.8, 13.5 per 1000) [83]. Even among those with 2 of the minimum required 3 prevalent factors for metabolic syndrome diagnosis, inverse multivariable-adjusted relative risks were evident with greater CRF (Fig. 5, Ref. [83]) suggesting that adequate fitness in mid-life might be especially effective in preventing later development of metabolic syndrome the prevalence of which rises sharply with age [84]. Additional corroboration comes from aerobic exercise training studies that have shown significant contemporaneous improvements in $\dot{V}\,O_{2\,m\,a\,x}$, CVD risk factors, and metabolic syndrome prevalence among middle-aged adults [85, 86, 87].

### 4.2 CRF and Subclinical Atherosclerosis

**Fig. 6.**
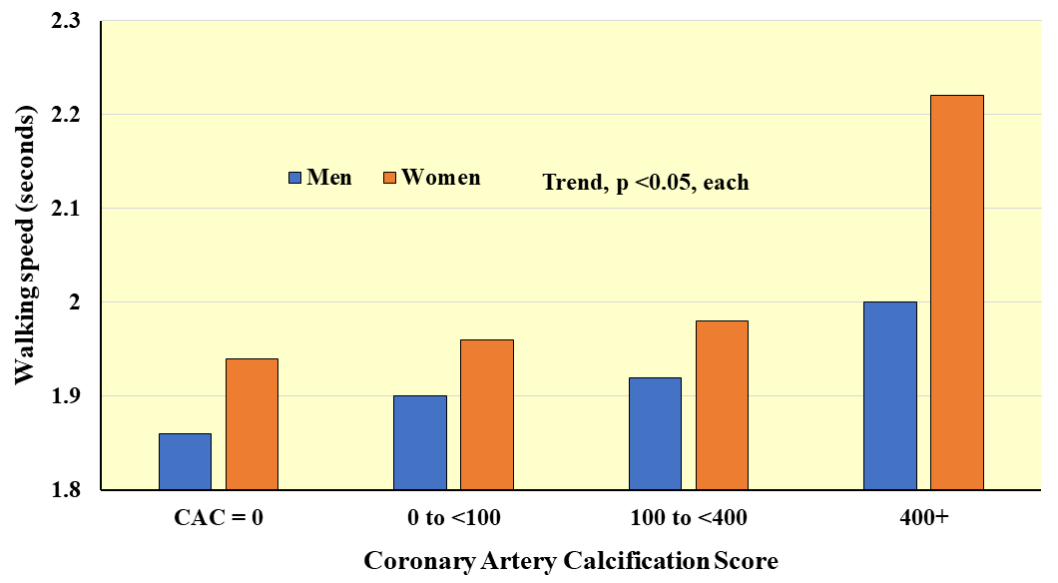
**Average walking speed over 8 feet according to coronary arterial calcification score**. *Adapted from Hamer M et al., Heart. 2010; 96(5): 380–384. [95]. *

The ability to characterize atherosclerotic CVD while in its subclinical stage provides new opportunities for arresting disease progression and preventing clinical CVD events [88]. Several measures of subclinical disease have been used in epidemiological and clinical investigations, some of which have been evaluated against CRF levels. Higher CRF is associated with fewer resting and exercise electrocardiographic indicators of obstructive coronary atherosclerosis in asymptomatic adults [25, 47, 89]. An extensive analysis on 3722 Korean men, ages 40 and older without clinical CVD, included measurements of $\dot{V}\,O_{2\,m\,a\,x}$, brachial-ankle pulse wave velocity (arterial compliance), carotid intima-media thickness (IMT), and coronary artery calcification (CAC; Agatston score) [90]. Each 1-MET higher CRF was associated with a 23% (*p *< 0.001) greater multivariable odds ratio for a composite healthy vascular outcome variable. Inverse associations between CRF and the heathy vascular outcome were seen over ages 40–49, 50–59, and ≥60 years, and in subsets of men with CAC >100 and IMT >0.8 mm. A cross-sectional study on 7300 German adults (mean 46) without CVD showed significant inverse gradients in mean IMT across incremental quartiles of measured $\dot{V}\,O_{2\,m\,a\,x}$ in both women and men [91]. Likewise, prospective studies have shown significant inverse associations between mid-life CRF and future IMT values indicative carotid artery disease [92, 93]. CRF is significantly associated with presence of any CAC (CAC >0), and a 41% lower multivariable-adjusted odds ratio when comparing the highest and lowest tertiles of CRF [94]. Among older British and U.S. adults faster timed walk test scores were significantly inversely associated with CAC score (Fig. 6, Ref. [95]) and IMT values in both women and men [95, 96]. In a 6-year moderate-intensity aerobic exercise training study, significant improvements in $\dot{V}\,O_{2\,m\,a\,x}$ and IMT were observed among men who were without clinical CVD and not taking statin medication [97]. A 4-year study on women in the menopausal transition similarly showed that aerobic exercise training significantly slowed IMT progression [98]. Collectively, the above findings suggest that fitness levels are correlated with subclinical atherosclerosis, and in turn, increasing CRF could potentially limit the severity and progression of subclinical CVD.

### 4.3 CRF and Incidence of Clinical CVD Events

A large body of epidemiological evidence supports inverse associations between CRF and the incidence of several primary clinical CVD outcomes [1, 2, 3]. Additionally, randomized controlled trials have demonstrated that aerobic exercise training in medically managed patients with existing CVD is safe and efficacious in the secondary prevention of recurrent events and mortality [99, 100, 101]. Guidelines have been published regarding the type, amount, and intensity of PA required to improve CRF and clinical cardiovascular status in both primary and secondary prevention settings [13, 34].

#### 4.3.1 CRF and Primary CVD Prevention

**Fig. 7.**
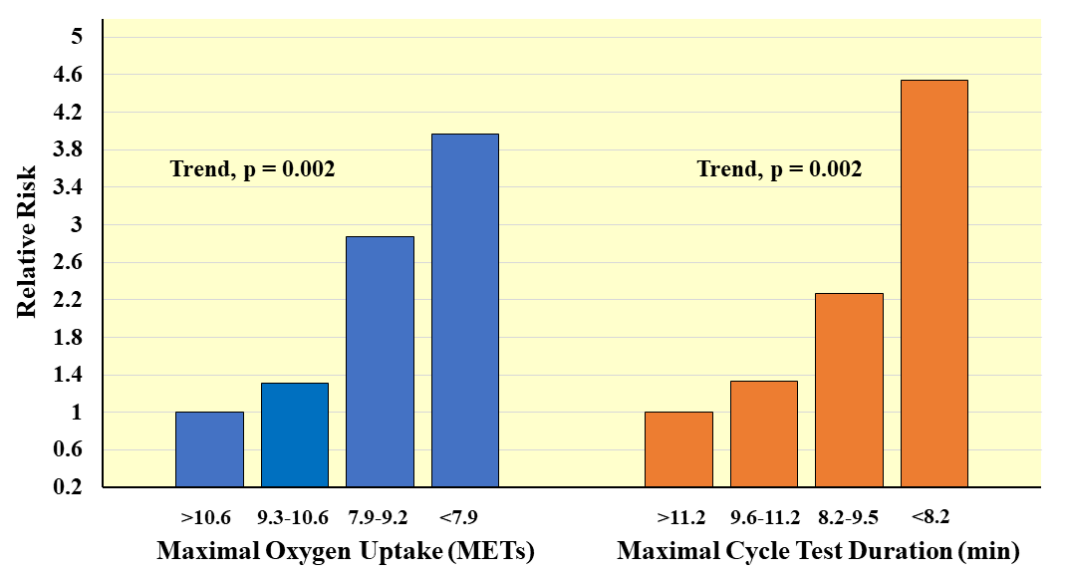
**Prospective association of measured maximal oxygen uptake and exercise test duration with CVD mortality in men. Relative risks adjusted for age and examination year**. *Adapted from Laukkanen JA, et al., Archives of Internal Medicine. 2001; 161: 825–831. [39]. *

CVD Mortality. The seminal work was contributed by Blair and coworkers who followed 13,344 adults ages 20–88 years for about 8 years after completion of a maximal treadmill fitness test and showed steep inverse gradients in age-adjusted rates of CVD mortality across incremental CRF tertiles in men (24.6, 7.8, 3.1 per 10,000; Trend *p *< 0.05) and women (7.4, 2.9, 0.8 per 10,000; Trend *p* = 0.09) [41]. The asymptote of the dose-response curve between CRF and all-cause mortality was 9 and 10 METs for women and men, respectively. Given that CVD accounted for the majority of deaths, it is likely that these same MET levels of CRF would be reasonable targets for primary CVD prevention, although many individuals might obtain benefit at even lower levels. In the Kuopio Ischemic Heart Disease and Risk Factor Study, 1294 Finnish men ages 40–60 years had their $\dot{V}\,O_{2\,m\,a\,x}$ measured and were then followed 10 years for CVD mortality [39]. Fig. 7 (Ref. [39]) shows the strong inverse association between measured $\dot{V}\,O_{2\,m\,a\,x}$ and CVD mortality, indicating a more than 3-fold higher mortality risk in men whose $\dot{V}\,O_{2\,m\,a\,x}$ was <7.9 METs compared to those with >10.6 METs. A separate investigation in the Finnish study showed each 1-MET increment in $\dot{V}\,O_{2\,m\,a\,x}$ was associated with a 22% (*p *< 0.001) lower multivariable-adjusted relative risk for sudden cardiac death [102]. In the Nord-Trondelag Norwegian cohort, each 1-MET increment in measured $\dot{V}\,O_{2\,m\,a\,x}$ was associated with a 17% (*p *< 0.05) and 12% (not significant) lower risk of CHD mortality in men and women, respectively [103]. A 20-year follow-up on 2994 women ages 30–80 who completed treadmill fitness testing as part of the Lipid Research Clinics Prevalence Study showed each 1-MET lower CRF was associated with a 17% greater (*p *< 0.01) multivariable-adjusted risk of CVD mortality [89], a magnitude of association for the same difference in CRF similar to men reported above and elsewhere [104].

Stroke Mortality. In an exceptionally large cohort of 1,166,035 Swedish men whose CRF was measured using maximal cycle ergometry at the time of entry into the military and who were followed 42 years for fatal stroke, the multivariable relative risks (95% CI) in the lowest and middle CRF tertile were 1.62 (1.35, 1.93) and 2.52 (1.82, 3.50), respectively, compared to high CRF [105]. Multivariable-adjusted relative risks for stroke mortality across incremental quartiles were 1.00, 0.47, 0.59, 0.50, Trend *p* = 0.004 in men and 1.00, 0.71, 0.62, 0.43, Trend *p* = 0.09 in women who completed maximal treadmill fitness testing in mid-life and were followed 17 years thereafter [106].

Non-fatal CVD. The vast majority of investigations on CRF and CVD have evaluated fatal events as the study outcome. However, the role of CRF in development on nonfatal clinical events is a critical piece of the primary prevention framework. A 10-year follow-up subsequent to maximal treadmill fitness testing showed significant inverse multivariable relative risks over tertiles of CRF for nonfatal total CVD (1.00, 0.89, 0.75, *p* = 0.001), CHD (1.00, 0.89, 0.76, *p* = 0.001), MI (1.00, 0.87, 0.73, *p* = 0.02), and stroke (1.00, 0.90, 0.71, *p* = 0.04) in 20,728 middle-aged men [25]. Among 5909 women in this study, inverse associations between CRF and each nonfatal endpoint were observed but did not achieve statistical significance due to the relatively small number of case counts. In Finnish men, each 1-MET increment in measured $\dot{V}\,O_{2\,m\,a\,x}$ was associated with relative risks of 0.87 (*p *< 0.001), 0.90 (*p* = 0.002), and 0.75 (*p *< 0.001) for nonfatal MI, stroke, and heart failure, respectively [107].

**Fig. 8.**
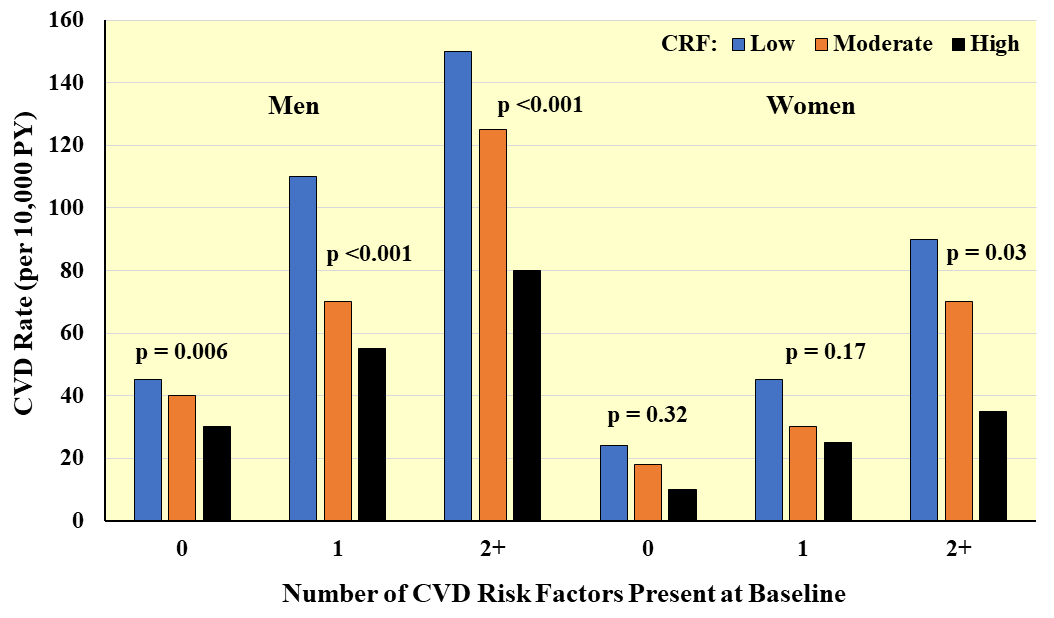
**Prospective association between CRF and CVD incidence according to number of major CVD risk factors present at baseline**. Rates are adjusted for age and examination year. PY, person-years. *Adapted from Sui X et al., American Journal of Epidemiology. 2007; 165: 1413–1423. [25].*

**Fig. 9.**
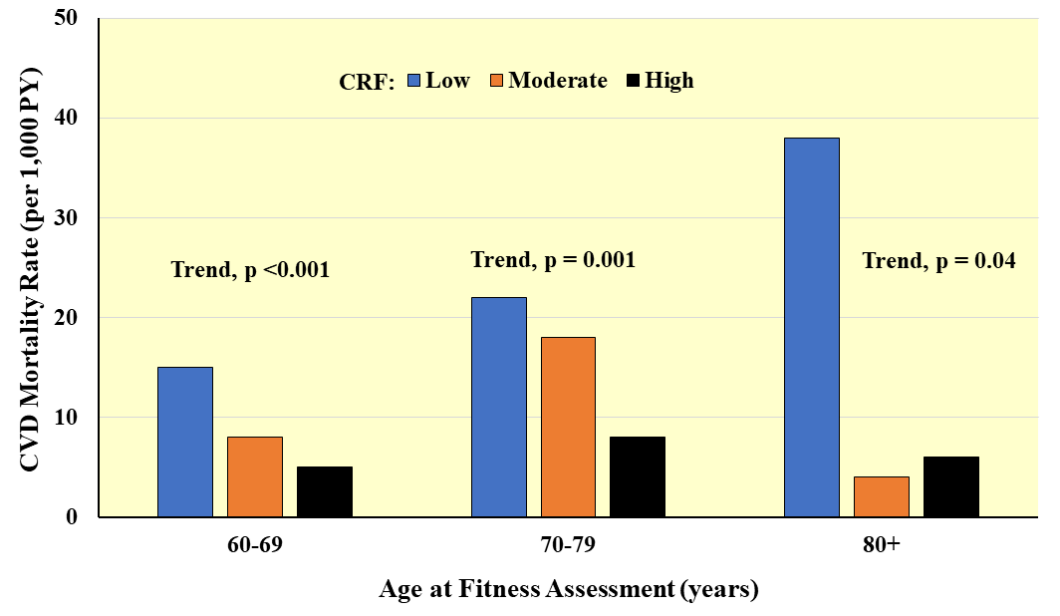
**Prospective association between CRF and CVD mortality according to age at baseline**. Rates are adjusted for sex and examination year. PY, person-years. *Adapted from Sui X et al., Journal of the American Geriatrics Society. 2007; 55: 1940–1947. [112].*

Population Subgroups. The protective association between CRF and clinical CVD events also is apparent in higher risk clinical subgroups. In 40,718 men without CVD, significant inverse associations between CRF and CHD mortality were observed in categories of <100, 100–129, 130–159, 160–189, and ≥190 mg/dL of fasting low-density lipoprotein cholesterol [108]. In women and men with 2 or more coexisting major CVD risk factors, higher CRF is associated with lower rates of nonfatal CVD events (Fig. 8) [25]. Among men with type 2 diabetes the 15-year cumulative probability of CVD mortality was substantially higher at 20% in those who were obese (BMI ≥30) compared to 10% in those with normal weight (BMI <25) [109]. However, in a multivariable-adjusted analysis that controlled for fasting glucose concentrations, the relative risk (95% CI) among obese men with moderate/high CRF was 1.5 (0.6, 3.6) and not statistically significant, whereas among men with low CRF it was 2.8 (1.4, 5.6), *p *< 0.01. Among men with normal weight, relative risks in men with high, moderate, and low CRF were 1.00, 2.3, 2.7, Trend *p *< 0.001. These results suggest that higher CRF might mitigate some of CVD risk conferred by type 2 diabetes including in those who are obese. Even in men with prognostically significant subclinical coronary atherosclerosis defined by CAC ≥400, the multivariable relative risk of combined fatal and nonfatal CHD was 0.23 (*p *< 0.01) in those with ≥10 MET compared to <10 MET levels of CRF [110]. The absolute risk of CVD events increases and CRF decreases with age [7, 27], thus with population aging the burden of CVD attributed to low CRF will increase. Strategies to maintain healthy CRF levels in later life could be an important risk reducing strategy. Among 1789 older adults enrolled in the Rancho Bernardo aging cohort who completed a treadmill fitness test, lower CRF was associated with a 72% (*p *< 0.05) greater risk of CHD mortality [111]. In another study on 4060 adults ≥60 years, strong inverse associations were observed between CRF and rates of CVD mortality within 10-year age categories (Fig. 9) [112]. Among those in the oldest age group (≥80 years), each 1-MET increment in CRF was associated with a 67% (*p* = 0.03) lower multivariable-adjusted CVD mortality risk.

Alternative CRF Indices. Several indicators of the hemodynamic and autonomic response to exercise also have been associated with CVD risk. These measures include insufficient increase in heart rate during exercise (chronotropic incompetence) and a slow heart rate recovery following exercise, and abnormal blood pressure responses during and after exercise. A prospective study on 1910 male veterans showed that failure to achieve at least 80% of age-predicted maximal heart rate during treadmill exercise testing was associated with a 2.8-fold (*p *< 0.001), whereas heart rate decreases of ≤22 beats at 2 minutes of recovery post-exercise was associated with a 2-fold (*p* = 0.02), higher risk of CVD mortality [113]. Men with both abnormalities had more than a 4-fold (*p *< 0.001) elevated CVD risk. Use of beta-blockers did not reduce the strength of these associations. Among 10,323 women and men without known CVD, compared to those achieving >99% of age-predicted maximal heart rate, the relative risk of incident CVD events was 1.24 (*p* = 0.02) and 1.61 (*p *< 0.001) in those achieving 96.6%–98.8% and 60.5%–96.5%, respectively, suggesting that additional risk might be harbored in adults with even modest chronotropic reductions during maximal effort [114]. A hypertensive response during maximal cycle ergometry testing was associated with a 34% (*p *< 0.05) and 19% (*p *< 0.05) higher risk of stroke and CVD, respectively [115], whereas each 100 mmHg higher systolic blood pressure at 2 minutes after maximal exercise was associated with a 7% (*p* = 0.001) greater risk of MI [116] in studies on men without known CVD at the time of testing.

#### 4.3.2 CRF and Secondary CVD Prevention

**Fig. 10.**
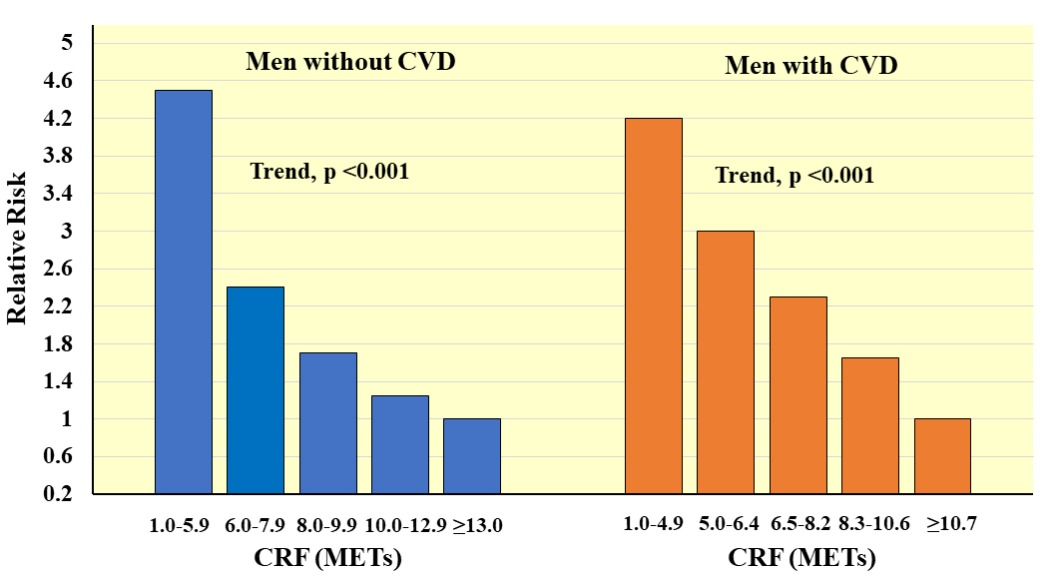
**Prospective association between CRF and all-cause mortality in men with and without CVD**. Relative risks are adjusted for age. *Adapted from Meyers J et al., New England Journal of Medicine. 2002; 346: 793–801. [42].*

Higher CRF in individuals who already have had a clinical CVD event is an important prognostic factor. In both women and men completing supervised cardiac rehabilitation following a clinical coronary event, $\dot{V}\,O_{2\,m\,a\,x}$ ≥3.7 METs in women and ≥4.3 METs in men was associated with 40–60% (*p *< 0.001) lower relative risks of mortality from all-causes and from cardiac causes [117, 118]. Further contribution in this area of work was made by Myers *et al*. [42] in their large prospective study on men with existing CVD who completed maximal treadmill tests at the Palo Alto Veteran’s Affairs Hospital. A steep inverse gradient in age-adjusted relative risks of all-cause mortality across incremental quintiles of CRF was observed, with more than a 4-fold (*p *< 0.05) greater risk in men with 1.0–4.9 METs compared to ≥10.7 MET levels of CRF (Fig. 10, Ref. [42]). While maximal CRF was lower than in men without CVD, overall, each 1-MET increment in CRF was associated with a 9% (*p *< 0.001) lower mortality risk in this secondary prevention cohort. In another cohort of men completing maximal cycle tests soon after an uncomplicated coronary event, each 1-liter/min increment in $\dot{V}\,O_{2\,m\,a\,x}$ was associated with 57% and 71% lower risks of all-cause and CVD mortality, respectively [40]. Even in men with prognostically relevant reductions in left ventricular function (ejection fraction (EF) <40%) after ST-elevation MI, achieving ≥4 METs on a maximal cycle ergometry test was associated with significantly lower all-cause mortality at 2- and 5-years post-testing [119]. The benefit of higher CRF in heart failure patients is not mitigated by beta-blocker use. Among heart failure patients with a mean left ventricular ejection fraction of ≤20%, each 1-mL $O_{2}$ •${\mathrm{kg}}^{-1}$•$\min^{-1}$ was associated with a 26% and 13% higher risk of all-cause mortality with and without beta-blocker use, respectively [120]. The significance of higher $\dot{V}\,O_{2\,m\,a\,x}$ in relation to enhanced prognosis in heart failure patients is seen across a wide range of age. CRF greater than 10 mL $O_{2}$ •${\mathrm{kg}}^{-1}$•$\min^{-1}$ is associated with 13%, 15%, and 16% lower mortality (*p *< 0.001, all) in heart failure patients aged ≤45, 46–64, and ≥65 years, respectively [121]. Two large randomized controlled exercise training trials have shown clear and strong evidence that moderate volumes and intensities of PA can improve CRF in medically managed heart failure patients with reduced ejection fraction [122, 123].

#### 4.3.3 Changes in CRF and CVD Outcomes

Studies evaluating longitudinal changes in CRF in relation to CVD outcomes provide a stronger test of the hypothesis than do those based on only a single assessment of CRF. Changes in CRF over two assessments are associated with significantly lower risks of developing major CVD risk factors including hypertension, diabetes, elevated cholesterol, and metabolic syndrome, to a large extent independent of changes in body weight [82, 124]. In a follow-up on 2014 men ages 40–50 at first of two maximal cycle ergometry assessments, the multivariable-adjusted relative risks over incremental quartiles of CRF change were 1.00, 0.64, 0.53, and 0.40 (*p *< 0.05, all) for incident ischemic stroke and were 1.00, 0.61, 0.55, and 0.49 (*p *< 0.05, all) for mortality [125]. In another study on 9777 middle-aged men, each 1-minute improvement in maximal treadmill exercise time over two assessments (Balke-Ware protocol) was associated with an 8% lower (*p* = 0.03) multivariable-adjusted risk of CVD mortality [126]. Because CVD risk in the above studies is based on change in CRF, it is less likely that misclassification bias is the primary explanation of the favorable associations with CRF reported in each study.

## 5. Is CRF More Important than PA?

**Fig. 11.**
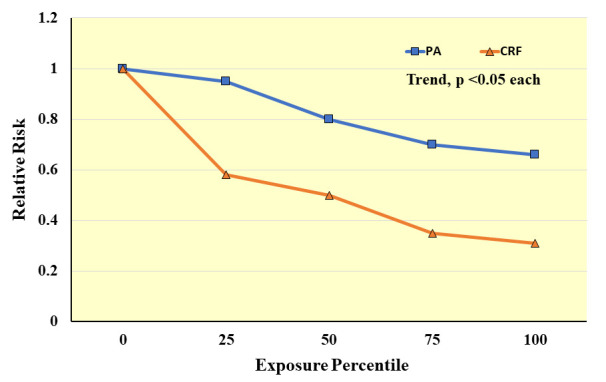
**Meta-analysis results of observational studies on cardiorespiratory fitness (CRF) or physical activity (PA) exposures in relation to the relative risk of clinical CVD events**. Exposure percentiles are ranked lowest (0) to highest (100) on the x-axis. *Adapted from Williams PT. Medicine & Science in Sports & Exercise. 2001; 33: 754–761. [127].*

One argument for CRF being a better reflection of exposure to sedentary lifestyles than self-reported or device-measured PA is less misclassification due to reporting biases and incomplete assessment of PA behavior [12]. Because CRF represents an integrated response in several biological systems, including genetics, required to support PA at given levels of effort, CRF might offer a broader representation of the underlying construct at a physiological level. A meta-analysis on observational studies that related either CRF or PA with incident CVD events showed that for both CRF and PA exposures there was a significant inverse pattern of association with CVD risk (*p *< 0.05, each), but the strength of association for CRF was far greater for CRF than PA, particularly at the lowest end of the exposure distributions where PA measurement precision tends to be poor (Fig. 11) [127].

Few investigations have included assessments of both CRF and PA in the *same study group*. In adults ages 18–95 years, $\dot{V}\,O_{2\,m\,a\,x}$ and PA measured by self-report or device are modestly correlated (r = 0.12–0.33) with stronger correlations evident when considering vigorous intensity PA [128, 129]. In a cohort of men referred for clinically indicated maximal treadmill testing, self-reported physical inactivity and low CRF (<5 METs) were associated with relative risks for mortality of 1.23 and 2.98 (*p *< 0.05, each) [8]. In a large cohort of 498,135 British adults, each 5 MET-hr/wk lower amount of self-reported PA was associated with a 1% greater mortality risk whereas each 1-MET lower CRF was associated with an 8% lower risk (*p *< 0.001, each) [130]. When stratified on tertiles of CRF, greater PA was associated with lower mortality risk only among those in the lowest and middle CRF tertile. Among Finnish men, the relative risk of incident MI was 0.32 (*p *< 0.001) for a 1-L/min higher $\dot{V}\,O_{2\,m\,a\,x}$ and 0.78 (*p* = 0.01) for a 1-hr/wk higher amount of self-reported conditioning exercise [131]. In 31,818 men and 10,555 women from the U.S., the multivariable-adjusted relative risks for mortality associated with self-reported inactivity, insufficient activity, and recommended activity were 1.00, 0.91, 0.87, Trend *p* = 0.07 in men and 1.00, 0.92, 0.83, Trend *p* = 0.52 in women [132]. Adding CRF to the model attenuated the associations essentially to the null. Corresponding associations across incremental tertiles of CRF with adjustment for PA, were 1.00, 0.64, 0.55, Trend *p *< 0.001 in men, and 1.00, 0.62, 0.61, Trend *p* = 0.02 in women. While direct comparison of CRF and PA exposures in relation to health risks is challenging for many reasons, the available data provide a fairly clear indication that CRF carries a stronger association than PA for a given clinical outcome when measured in the same group of individuals. Promoting PA at levels sufficient to enhance or maintain healthy CRF [13, 18] is likely to correspond with better cardiovascular health.

## 6. Should CRF be a Component of Individual-Level CVD Risk Assessment?

In the preceding sections it was clear that CRF is associated with one’s propensity for adverse CVD outcomes. In an office setting, healthcare providers typically rely on multiple risk factor scoring algorithms to determine their patient’s short-term probability of a clinical CVD event and, in turn, guide decisions on initiation and intensity of primary preventive measures [7]. A small number of studies have attempted to quantify the additional prognostic value of adding a measure of CRF to conventional office-based CVD risk calculation (e.g., Framingham Risk Score). Table 3 (Ref. [133]) summarizes results of a study on 41,708 men who were without clinical CVD at the time of baseline examination that included a maximal treadmill fitness test [133]. After 17 years follow-up, each 1-unit increment in Framingham risk score (10-Year predicted probability) was associated with a 6% higher relative risk of CVD and CHD mortality (*p *< 0.05, each). When CRF was added to the regression model, the relative risks of each outcome associated with the Framingham score attenuated to 1.03 (*p *< 0.05, each), and the relative risks for a 1-MET decrement in CRF were 1.24 and 1.27 (*p *< 0.05, each). When men were grouped on clinical categories of Framingham score (<10%, 10–20%, >20% 10-Year probabilities), in all categories the relative risks for CVD and CHD mortality were significantly increased with each 1-MET lower CRF. These findings suggest that clinical CVD risk assessment should not end with assessment of traditional modifiable risk factors, but instead should also include assessment of CRF. Similar findings have been reported in other cohort studies [134, 135, 136] and the issue of how to incorporate CRF assessment into office practice has been discussed in an American Heart Association pronouncement [137].

**Table 3.** **CVD mortality according to framingham risk score and CRF in men**.

		CVD mortality		CHD mortality
		(1307 deaths)		(792 deaths)
FRS alone*				
	FRS (per 1-unit increment)	1.06 (1.04, 1.07)		1.06 (1.05, 1.08)
FRS plus CRF*				
	FRS (per 1-unit increment)	1.03 (1.02, 1.06)		1.02 (1.01, 1.06)
	CRF (per 1-MET decrement)	1.24 (1.21, 1.27)		1.27 (1.22, 1.32)
Likelihood ratio statistic		214.6 (*p *< 0.001)		165.7 (*p *< 0.001)
		Framingham risk score (10-year probability)		
		<10%	10–20%	>20%
		(Low risk)	(Intermediate risk)	(High risk)
CVD ${\mathrm{death}}^{†}$				
	CRF (per 1-MET decrement)	1.21 (1.15, 1.27)	1.15 (1.10, 1.22)	1.18 (1.10, 1.25)
CHD ${\mathrm{death}}^{†}$				
	CRF (per 1-MET decrement)	1.21 (1.12, 1.28)	1.22 (1.14, 1.29)	1.16 (1.08, 1.27)

Data are hazard ratio (95% confidence interval). *Model also includes age, examination year, and family history of CVD. †Model also includes age, examination year, family history of CVD, abnormal electrocardiogram, chronotropic incompetence. *Adapted from LaMonte MJ et al., Circulation. 2005; 112(Suppl II): II-829. [133].*

## 7. Limitations

The overview presented here on CRF and CVD prevention was not an exhaustive review of the published scientific literature nor did it address all possible mechanisms by which greater CRF might enhance cardiovascular health. The exemplar studies discussed were selected to make specific points but may not represent the range of available findings in a given area. Future studies that include both a performance-based measure of CRF and a well-documented assessment of PA would be helpful to clarify the extent to which PA and CRF confer independent cardiovascular benefits, especially in older adults whose maximal CRF is limited. Continued efforts to identify an absolute level of CRF where CVD risk reduction would be expected in apparently healthy adults, and to identify the PA dose required to achieve that level of CRF, is critical to enhancing future public health recommendations on lifestyle behaviors.

## 8. Conclusions

As depicted conceptually in Fig. 3 and supported by evidence summarized herein, CRF is a modifiable factor associated with multiple paths in CVD incidence and prognosis. The gold standard measure of CRF is the maximal oxygen uptake ($\dot{V}\,O_{2\,m\,a\,x}$). Because differences in $\dot{V}\,O_{2\,m\,a\,x}$ between individuals is due largely to differences in maximal cardiac output, $\dot{V}\,O_{2\,m\,a\,x}$ is a clinical indicator of cardiac function. Not surprisingly, numerous studies have shown that CRF assessed with maximal exercise testing is strongly associated with major CVD risk factors, left ventricular structure and function, coronary and peripheral arterial compliance, and measures of subclinical atherosclerosis. Studies also have shown that CRF adds prognostic value to established multifactor CVD risk prediction models, which are the cornerstone in office-based individual-level risk assessment and prevention. Moderate intensities and volumes of regular PA can improve CRF in both healthy and diseased adults. Because CRF is measured more objectively thsan PA, and because CRF might better reflect the influences of both behavior and genetics on functional status, CRF might be a more accurate indicator of the consequences of a sedentary or irregularly active lifestyle. A public health imperative in this century is to aggressively promote at the population level PA that is sufficient enough to enhance and maintain CRF, and in turn, for healthcare providers to routinely assess and monitor their patients CRF level as done with other clinical vital signs.
